# Canadian French translation and linguistic validation of the child health utility 9D (CHU9D)

**DOI:** 10.1186/s12955-018-0998-4

**Published:** 2018-08-29

**Authors:** Thomas G. Poder, Nathalie Carrier, Harriet Mead, Katherine J. Stevens

**Affiliations:** 1 UETMIS and CRCHUS, CIUSSS de l’Estrie – CHUS, Sherbrooke, Canada; 2 CRCHUS, CIUSSS de l’Estrie – CHUS, Sherbrooke, Canada; 3 ICON Language Services, Sherbrooke, Canada; 4 The University of Sheffield, Sheffield, England

**Keywords:** Canadian French translation, Linguistic validation, Child health utility 9D, Cost utility analysis, Pediatric, Forward and back translation

## Abstract

**Background:**

Several preference based measures are validated for adults in cost utility analysis, but less are available for children and many researchers have criticized the quality of pediatric economic studies. The objective of this study was to perform a Canadian French translation and linguistic validation of the Child Health Utility 9D (CHU9D) that was conceptually equivalent to the original English version for use in Canada.

**Methods:**

The translation and linguistic validation were realized by ICON Clinical Research (UK) Limited in association with the developer of the CHU9D and Canadian collaborators. This was done in accordance with industry standards and the guidance of the Food and Drug Administration (FDA) for patient-reported outcome (PRO) instruments. Five steps were considered: concept elaboration; forward translation; back translation; linguistic validation; proofreading and final verification.

**Results:**

The CHU9D Canadian French translation and linguistic validation were realized without any major difficulties. Only 3 changes were made after the forward translation and 5 after the back translation. The result of back translation was very similar to the original English version. Six additional changes suggested by the developer team were accepted and the linguistic validation with five children led to 2 additional changes. Most changes were generally to change one word to better sounding Canadian French.

**Conclusion:**

We produced a Canadian French translation and cross-cultural adaptation of the Child Health Utility 9D (CHU9D). Before being used in clinical settings and research projects, the final Canadian French translation needs to be validated for metrological qualities of reliability and validity.

**Electronic supplementary material:**

The online version of this article (10.1186/s12955-018-0998-4) contains supplementary material, which is available to authorized users.

## Background

For several years, the use of economic evaluation to inform and aid health-care decisions has been increasing [1, 2]. A method frequently used is cost-utility analysis [3]. In this method, the health outcome is expressed in terms of a “utility based” unit of measurement. Health utility is a term used by health economists to refer to the subjective level of wellbeing that people experience in different health states. The most widely used measure of health outcome in cost-utility analysis is the quality adjusted life year (QALY). The QALY combines length of life and quality of life into a single measure [4]. Generic preference-based measures of health-related quality of life (HR-QoL) can be used to obtain information on quality of life to use in calculating QALYs [5].

There are several generic preference-based measures validated and used for adults [6], but less are available for children [7]. Moreover, several researchers have criticized the quality of pediatric economic studies, particularly the lack of QALY instruments specific to children [8–10]. To date, the main instruments available for children are: the European Quality of life 5-Dimension Youth version (EQ-5D-Y) [11], the Assessment of Quality of Life 6-Dimension (AQoL-6D) [12], the Health Utilities Index 2 (HUI2) [13, 14], the CHSCS-PS [15], and the Child Health Utility 9D (CHU9D) [16–18]. Other instruments were also developed but less used [7], such as the 16-D and 17-D [19, 20].

The EQ-5D-Y has been adapted from the EQ-5D-3 L (a generic preference-based measures for adults, widely used and recommended by NICE in its reference case [21, 22]) and has kept the same dimensions of health as the adult version, wording it for children’s comprehension, which remains questionable [9, 11]. In addition, no preference weights (i.e., a set of utility values that reflects the preferences people have for different health states described by different dimensions of quality of life) data have been developed for the EQ-5D-Y. The AQoL-6D was developed for adults and was adapted in the sense that preference weights are available for use in adolescents [12]. The Health Utilities Index 2 (HUI2) is used in practice and is well validated [14] but the dimensions are designed to assess impairment and disability rather than the impact of these on the child’s quality of life and participation [17]. Also the preference weights for HUI2 are derived from parents and not children. The CHSCS-PS is based on the HUI2 and HUI3, but it was created only for preschool children (2 to 5 years) and needs to be responded by parents and/or nurses [15]. Actually, there is no preference weights developed for the CHSCS-PS. Finally, the CHU9D is the only instrument that has been developed with children with the aim of exploring how health status affects their quality of life [23]. It has been demonstrated to be acceptable, practical and valid for application with children and adolescents aged 7–17 years in two countries (UK and Australia). As with other preference-based measures, the CHU9D uses preference weights data from adults (UK), but also from adolescents (Australia). Since the CHU9D is only available in a few languages, there is a need to perform translation and linguistic validation in other contexts.

## Objective

The objective was to develop a Canadian French translation of the Child Health Utility 9D (CHU9D) that was conceptually equivalent to the original English version developed in the United Kingdom.

## Methods

In June 2017, our team contacted the developer of the original instrument to perform a Canadian French translation of the Child Health Utility 9D (CHU9D) for use in Canada. The translation was carried out in collaboration with the developer by ICON Clinical Research (UK) Limited, a professional translation company who specialises in translating utility measures. This company is in accordance with industry standards [24, 25] and the guidance of the Food and Drug Administration (FDA) for patient-reported outcome (PRO) instruments [26].

An experienced project manager coordinated the project (B.A. in applied languages). The tasks of the project manager were to select all translators and a linguistic validation consultant (LVC) (B.A. in political science), to check if translators performed their tasks in the correct manner, and to manage the project so that the schedule was maintained. The project manager also worked with the LVC during each stage of the process to hone the translation where necessary. At each step, the developer of the instrument and a Canadian collaborator (Ph.D. in health economics) were involved and provided suggestions to improve the quality of the translations, in accordance with the meaning of the original version in English.

The Child Health Utility 9D (CHU9D) is a generic measure of health-related quality of life for children aged 7–17 years. It consists of 9 dimensions, including worried, sad, pain, tired, annoyed, schoolwork, sleep, daily routine, and ability to join in activities. Within each dimension, there are 5 different levels indicating increasing levels of severity. At present there is the original UK English version and Chinese, Spanish, Welsh, Dutch, Italian, Japanese, and Danish translations available. More information on the CHU9D is available on the website of the University of Sheffield [27]. For the translation in French Canadian, all questions and instructions were devised in 69 words or sentences (i.e. some sentence consisted only in one word).

### Translation and validation

The five steps of the translation were: concept elaboration; forward translation; back translation; linguistic validation; proofreading and final verification.

#### Concept elaboration

In order to aid the forward translators in choosing the right terminology for conveying the same meaning as the source, a concept elaboration document was produced and shared. The collaboration of the instrument developer was also confirmed at this stage.

#### Forward translation

Two translators developed a forward translation each. One of the translators has a B.A. in political science, and the other a B.A. in chemistry. Both were French native speakers and fluent in English, and had extensive experience in the translation of PRO instruments. The two forward translations were reconciled into a third translation by the LVC, which was then sent to an independent linguist (M.A. in translation theory and practice) who had not yet been involved with the project. As with the LVC and the two previous translators, the linguist was a French native speaker fluent in English. He was asked to check the translation for errors of spelling, grammar, punctuation and typography, as well as to check the translation against the English source in order to ensure that no text had been mistakenly omitted or repeated. The linguist also reviewed all aspects of the translation, which included terminology choice, style, typos, and formatting errors. Finally, the LVC gave feedback on changes suggested by the independent linguist.

#### Back translation

Two back translations of the reconciled translation were performed. One of the back translator was a native English for Canada and had diplomas in ‘Anatomy of a Clinical Trial Protocol: Important Concepts and Essential Terminology for Accurate Translation’ and ‘Clinical Trials and Medical Documentation: Resources and Strategies for New Translators’, while the other was a native English for UK and had a B.A. in communications. Both translators were fluent in French and English and had never previously been involved on a project with the CHU9D. The project manager reviewed the back translations and provided recommendations and comments to the LVC. The latter refined the translation to correct any inconsistencies or errors. The back translation report was then submitted to the instrument developer and Canadian collaborators (called the “developer team” thereafter for simplicity) who reviewed the decisions made up to and including this stage of the translation process. Any comments or questions from the developer team were discussed with the project manager and the LVC until a satisfactory resolution was found.

#### Linguistic validation

A series of individual, face-to-face interviews were conducted. All respondents were asked to complete a copy of the questionnaire before to answer a series of open-ended questions about each instruction, question and response option. These questions were: “Do you understand this instruction/item/response scale?; If there are any difficulties, how would you reword this instruction/item/response scale?; What does this item mean to you? (Respondents were asked to rephrase the item using their own words); Are the response options consistent with this item?” See Additional file 1 for a full list of questions. Interviewers could record the interviews to help with their notes if they wished to do so and provided a transcription to the LVC. All responses were summarized by the LVC in a single report. This report also included suggestions and comments related to the translation that were provided by the respondents during the interviews. The project manager reviewed the report and discussed with the LVC about any issues arising until a satisfactory resolution was found. The developer team reviewed the decisions and discussed any points with the project manager and the LVC until a consensus was found.

#### Proofreading and final verification

A proof reader (M.A. in translation) external to the project was recruited to check the translation for errors of spelling, grammar, punctuation and typography, as well as to check the translation against the English source to ensure that no text had been mistakenly omitted or repeated. This proof reader was a native speaker of Canadian French fluent in English. Finally, the LVC provided feedback on changes suggested by the proof reader.

## Results

### Forward translation

Results of the two forward translations are presented in Table 1. In the reconciliation process, there were 18 (26.1%) sentences that were the same, 48 (69.6%) where one of the two translations was selected (34 and 14 for first and second translator, respectively), and 3 which were the result of a combination of the 2 translations.

**Table 1:** Results of forward translation

	*n* (%) of sentence	Decision with manager and LVC
Forward translation reconciliation		
- Same translation	18 (26.1)	
- Used result of one of translator	48 (69.6)	
Translator1/Translator2	34/14	
- A combination of the 2 translations	3 (4.3)	
Editing changes (*n* = 7)	17 (24.6)	
- Just changed the tag	1	Accepted
- Reviewer was not sure that the translation of “upset” by “troublé” will be understood by young child	1	‘troublé’ was kept to be different from ‘annoyed’
- Add masculine/feminine in the section title for “Inquiet/Inquiète”, “Fatigué(e)” and “Contrarié(e)”	3	Refused. The LVC thought it was fine to just keep the title in the masculine. Related statements were already masculine/feminine.
- Change “Devoir” by “Travaux/devoirs”	6	Accepted
- Suggest “Sommeil” instead of “dormir”	1	Refused. The LVC considered that “dormir” would be better for children
- Replace “aucun mal à dormir” by “aucune difficulté à dormir”	4	Accepted
- Change “tes” by “ses”	1	Refused. The LVC disagreed with the changes since this change went against the original version.

The reconciled translation was sent to an independent linguist who had not previously worked on the translation project. Of the 69 sentences, there were 17 (24.6%) suggestions of change, but only 7 different since some suggestion came back several times. The project manager and the LVC accepted 11/17 (3/7) changes. Accepted changes were to change the tag in one situation, to change “Devoir” by “Travaux/devoirs” and replace “aucun mal à dormir” by “aucune difficulté à dormir”. Rejected suggestions were to have masculine/feminine in the title and to change “your” by “his”. The reviewer was not sure that the translation of “upset” by “troublé” will be understood by young children, but the original translation was kept to be different from ‘annoyed’.

### Back translation

Two new translators completed the back translation of the reconciled translation (Table 2). In the first internal validation of the back translations, no changes were necessary in 56 (81.2%) of cases and the original translation was used. The back translations were identical or nearly identical to the original English version for 22 (31.9%) and 9 (13%) of sentences respectively. Twenty-five (36.2%) needed a discussion between the LVC and the project manager for further clarification to finally accept the original translation. Finally, only 13 modifications (6 different) were suggested and 12 (5 different) were accepted.

**Table 2:** Results of back translation

	*n* (%) of sentence	Decision with manager, LVC and developer team
Back translation, manager and LVC review		
- Identical translation	22 (31.9)	
- Almost identical translation	9 (13.0)	
- Not the same translation, but after discussion, kept the original translation	25 (36.2)	
- Modifications (*n* = 6)	13 (18.8)	
- Unbold “seule” in the sentence	1	Accepted
- Removed “du tout” in the sentence	5	Accepted
- Replace “mal” by “douleur”	4	Accepted
- Replace “l’étude” by “faire ses leçons”	1	Accepted
- Replace “Participer à” by “Capable de participer à”	1	Accepted
- Add “activités que je veux”	1	Developer refused the addition. The term “que je veux” was not in the original English version.
New changes of developer team review (*n* = 7)	18 (26.1)	
- Difficulties with title translation	1	Accepted new title
- Change “troublé” by “malheureux”	2	Accepted
- Replace “j’ai” by “je ressens”	5	Accepted
- Replace “douleur” by “avoir mal”	4	Refused by LVC and manager
- Change “difficulté à dormir” by “mal à dormir”	4	Accepted
- Replace “t’habiller” by “s’habiller”	1	Accepted
- Only bold text was changed	1	Accepted

The back translation report was submitted to the developer team. They suggested 18 new modifications (7 different), that required several exchanges between the LVC and the developer team. There was a lot of discussion about the translation of the title, and external input from another health economist fluent in French and English was also used to reach a consensus. In the other modifications suggested, one word was changed in three separate situations (11 sentences) to sound more natural in Canadian French (i.e. changed “troublé” by “malheureux”; “j’ai” by “je ressens”; “difficulté à dormir” by “mal à dormir”), a pronoun was changed in one case (i.e. “t’habiller” by “s’habiller”) and one word in bold was changed to non-bold format to better reflect the English original sentence. A Canadian collaborator also suggested changing “douleur” to “avoir mal” to achieve a more natural Canadian French, but the LVC refused and thought that “douleur” was more appropriate.

### Linguistic validation

The linguistic validation was conducted with 5 participants - 2 girls and 3 boys - aged between 7 to 17 years (mean 13.8; standard deviation 3.63), corresponding to a mix of healthy participants and participants with any condition (Table 3).

**Table 3:** Characteristics of children participating in the linguistic validation

	(*n* = 5)
Gender	
Girl	2
Boy	3
Age	
7–10 years	1
11–12 years	1
15–16 years	1
17 years	2
Profession of parents	
Military and businesswoman	2
Librarian and teacher parents	2
Engineer and teacher parents	1

Generally, the children understood each instruction, question and response (Table 4). Only two changes were made as a result of the children’s responses. The term “contrarié” which was the translation of “annoyed” was not well understood by the majority of children and was changed to “embêté”. The term “douleur”, the translation of “pain”, was misunderstood and changed by “avoir mal”, as previously suggested by the developer team. In the two cases, the modification was better understood by the children. In addition, when a Canadian collaborator reviewed children’s responses, he observed that there was 1 change, which appears in 5 sentences, that was accepted in the previous steps but was not changed in the version that the children responded to. After observing this problem, the questionnaire was changed. This question was retested in children and the new version was better understood. Finally, all respondents were comfortable with the questionnaire, clearly understood the Canadian French that was used, did not have any difficulty understanding the instructions and the response options made sense for them. They did not have any other suggestions to help improving the questionnaire.

**Table 4:** Results of the linguistic validation and review with the developer team

Children comprehension	*n* (%) of sentence	Second validation with children
- Good comprehension, no change	51 (73.9)	No new validation
- Error: The changes previously requested have not been made.	5 (7.2)	
- “Troublé” had to be changed by “Malheureux”	5	Better understood with new version
- Modifications	12 (17.4)	
- The term “douleur” was misunderstood and changed by “avoir mal”	6	Better understood with new version
- The term “contrarié” was misunderstood and changed by “embêté”	6	Better understood with new version

### Proofreading and final verification

The translation was sent for proofreading to an independent proof reader and no new changes were made. The final version of the CHU9D in Canadian French is now available upon request at the University of Sheffield [27].

## Discussion

This study reported the Canadian French translation of the CHU9D for use in Canada. This is the first translation of the CHU9D in French and could be used as a reference material for subsequent adaptation in other contexts where French is used. This is also the first publication of a cultural adaptation of the CHU9D from the original version in the United Kingdom. Although previous translation and linguistic validation were performed in other languages using the same methodology as in this study, no publication was done. The translation has been meticulously carried out by accredited professionals following the standard translation and linguistic validation methodology in accordance with industry standards and the FDA’s guidance for the PRO industry. This methodology ensures that the translation is conceptually equivalent with the original English version, harmonized with other translations and acceptable for inclusion in regulatory claims and IRB submissions [25].

The CHU9D Canadian French translation was realized without any major difficulties. The editing step after the forward translation required only 7 changes with 3 accepted. For the back translation, only 5 changes were proposed, but one was rejected by the developer team. The new translation was very similar to the original English version. Most modifications were generally to change one word to sound more natural in Canadian French.

One of the few points that has been problematic and that has necessitated many exchanges between the translation company and the developer team was the translation of the title. The translators suggested translating the title “Child Health Utility 9D” to “Children’s Health Status on 9D (CHU9D)” and to exclude the term “Utility” so that children would understand the title. However, utility is a term used by health economists and used in cost utility analysis and is well understood by those who use measures such as these in their research, so it was felt important to retain use of the term utility in the title. It makes no difference to whether children would be able to complete the questionnaire itself or not. The title was therefore translated as “Mesure de l’utilité reliée à la santé chez l’enfant (CHU9D)” as suggested by a Canadian collaborator and a health economist external to the project and agreed by the project team.

A major strength of this study was to include the instrument developer in the translation process. The advantage of including her in this process was to clarify any ambiguities and to clarify the concepts used [25]. Another strength was to document each change at each step in the process for more transparency. Currently, the vast majority of articles on the translation of health questionnaires present the results of the validation with regard to metrological properties rather than the process of translation and linguistic validation itself and the problems encountered. There are very few papers in the literature on the process of translation and this study is one of the few providing details for each step [28, 29]. This will help in the aim to remind that the translation process is as much important as the validation of metrological properties and must follow a rigorous process. Indeed, the translation and linguistic validation is a prerequisite for the use of PBM and allow different version of a same instrument to be equally natural and acceptable and to practically perform in the same way [30].

Finally, this study has two limitations. The first one is that it was completed before the publication of the new COSMIN methodology for evaluating the content validity of patient-reported outcome measures, especially as regard to comprehensibility [31]. This would have helped in the design of the study and probably have improved its quality. Although this study covers most of the criteria recommended by the COSMIN methodology, it would have been valuable to better consider the last item of comprehensibility proposed by the COSMIN methodology, that is to say if the response options match the question. As regards to the other criteria, these ones has been considered during the development of the CHU9D in the United Kingdom [16, 17]. The second limitation is that we performed the linguistic validation with only five children. It would have been more robust to interview more children. However, we used a criterion of saturation and we noted that the two last children interviewed did not report any more comments than the three first one. Thus explaining why we stopped at five children.

## Conclusion

We produced a French translation and cross-cultural adaptation of the Child Health Utility 9D (CHU9D). Before being used in clinical settings and research projects, the final French translation needs to be validated for metrological qualities of reliability and validity.

## Additional file

Additional file 1: Cognitive debriefing grid template. (DOC 120 kb)

## Abbreviations

AQoL-6D: Assessment of quality of life 6-Dimension.
CHU9D: Child health utility 9D.
EQ-5D-Y: European quality of life 5 dimension youth version.
FDA: Food and drug administration.
HUI2: Health utilities index 2.
HR-QoL: Health-related quality of life.
LVC: Linguistic validation consultants.
PRO: Patient-reported outcome.
QALY: Quality adjusted life year.
