# Supplementary material for: Canadian French translation and linguistic validation of the child health utility 9D (CHU9D)
Source: Health Qual Life Outcomes. 2018 Aug 29;16:168. doi: 10.1186/s12955-018-0998-4 (PMC6114803; doi:10.1186/s12955-018-0998-4)
Supplement: Supplementary file 1 — Cognitive debriefing grid template. (DOC 120 kb) [file 12955_2018_998_MOESM1_ESM.doc]

| **(1) ID** | **(2) source english text** | **(3) DRAFT TRANSLATION FOR TESTING** | **(4) probing questions / respondents’ answers** | **(5) comments & discussion** |
| --- | --- | --- | --- | --- |
| 1  d82e5a63-40ab-496b-a32c-5febc920de9a | Child Health Utility 9D | Mesure de l’utilité reliée à la santé chez l’enfant (CHU9D) | Do not test. |  |
| 2  f1cdeec0-4eb3-417c-a0fc-f592b30dc22f | Instructions | Instructions | What does ‘instructions’ mean to you?  *  *  *  *  * |  |
| 3  a98b0026-a171-4d59-a496-f9152f1fa5e7 | These questions ask about how you are **today.** | Ces questions te demandent comment tu vas **aujourd’hui**. | What are the questions going to ask about?  *  *  *  *  * |  |
| 4  c409c8d1-6269-495c-997f-02d6e5e4eb1e | For each question, read all the choices and decide which one is most like you **today**. | Pour chaque question, lis tous les choix et décide lequel est le plus proche de la façon dont tu te sens **aujourd'hui**. | What do you need to do for each question?  *  *  *  *  *  What day do you need to think about when answering the questions?  *  *  *  *  * |  |
| 5  74c6d702-5ad7-4d76-8be5-6f9f82abc00e | Then put a tick in the box next to it like this {MQ}. | Ensuite, coche la case à côté de la réponse comme ceci {MQ}. | What is this telling you to do?  *  *  *  *  * |  |
| 6  4c0b5ef7-4cc1-494a-858e-df03a0181134 | Only tick **one** box for each question. | Ne coche qu’**une** seule case pour chaque question. | How many boxes can you tick per question?  *  *  *  *  * |  |
| 7  9ef970d0-5c4e-49a2-81b0-d6b585ab016b | Example | Exemple | Can you explain what an ‘example’ is?  *  *  *  *  * |  |
| 8  cf08ada5-7e69-4091-9237-5c3065be59a0 | Today I feel quite upset so I will tick this box. | Aujourd'hui, je me sens assez malheureux/malheureuse, alors je vais cocher cette case. | Can you explain what feeling ‘upset’ means?  *  *  *  *  * |  |
| 9  6705d8ba-b998-4791-bc80-448d81248014 | **Upset** | **Malheureux** | Can the respondent give examples of times when they might be “upset”?  *  *  *  *  * |  |
| 10  7756000c-d4d7-4640-9520-b95d35f09c48 | I don’t feel upset today | Je ne me sens pas troublé(e) aujourd'hui | Looking at the scale on the thermometer, if ‘I don’t feel upset today’ is 0, what number would you give the other response options?  *  *  *  *  * |  |
| 11  c22fe9d7-516c-4c84-9ede-634fe184c87b | I feel a little bit upset today | Je me sens un tout petit peu troublé(e) aujourd'hui |  |
| 12  592dfdb3-5b4b-4e47-b1c6-3e70050e46ca | I feel a bit upset today | Je me sens un peu troublé(e) aujourd'hui |  |
| 13  6797685b-7b77-4f03-bc6a-032f0a98035e | I feel quite upset today | Je me sens assez troublé(e) aujourd'hui |  |
| 14  cc3ad92e-f1d1-4229-8de2-868c39437bbf | I feel very upset today | Je me sens très troublé(e) aujourd'hui |  |
| 15  03083f2c-83e4-4bc1-ac0c-d472d305c893 | Now think about and answer the rest of the questions below | Maintenant, réfléchis et réponds au reste des questions ci-dessous | What does ‘think about’ mean?  *  *  *  *  * |  |
| 16  bb776c17-cea9-43c3-98e3-47d6187e37ef | **Worried** | **Inquiet** | Can you explain what ‘worried’ means?  Can the respondent give examples of times when they might be “worried”?  *  *  *  *  * |  |
| 17  7f4a1c92-d8d5-4c08-890d-84f4fd2f33ea | I don’t feel worried today | Je ne me sens pas inquiet/inquiète aujourd'hui | Looking at the scale on the thermometer, if ‘I don’t feel worried today’ is 0, what number would you give the other response options?  *  *  *  *  * |  |
| 18  f9357099-5e12-4ac5-8a47-48bdf7aff4c8 | I feel a little bit worried today | Je me sens un tout petit peu inquiet/inquiète aujourd'hui |  |
| 19  036e56e9-95fd-488b-9048-64e5eb48a472 | I feel a bit worried today | Je me sens un peu inquiet/inquiète aujourd'hui |  |
| 20  8f413afe-d912-4f51-b8b5-bcdf79ec9f7d | I feel quite worried today | Je me sens assez inquiet/inquiète aujourd'hui |  |
| 21  9f62d41c-79fe-49ef-b5c2-d5e812d6598b | I feel very worried today | Je me sens très inquiet/inquiète aujourd'hui |  |
| 22  780ba755-6552-4fa4-934c-3027dc0bbb5b | **Sad** | **Triste** | Can you explain what ‘sad’ means?  *  *  *  *  *  Can the respondent give examples of times when they might be “sad”?  *  *  *  *  * |  |
| 23  4d17ffb1-77a8-4144-bbec-493d655bd3a5 | I don’t feel sad today | Je ne me sens pas triste aujourd'hui | Looking at the scale on the thermometer, if ‘I don’t feel sad today’ is 0, what number would you give the other response options?  *  *  *  *  * |  |
| 24  c5b7b458-9ec2-4a00-b9bb-627f1650cae1 | I feel a little bit sad today | Je me sens un tout petit peu triste aujourd'hui |  |
| 25  192dd08e-202c-4b56-89f6-4b96ed3f7030 | I feel a bit sad today | Je me sens un peu triste aujourd'hui |  |
| 26  0de3307b-c592-445d-8391-66f8540062df | I feel quite sad today | Je me sens assez triste aujourd'hui |  |
| 27  73436759-4aa1-4d3b-adda-be256e6aef34 | I feel very sad today | Je me sens très triste aujourd'hui |  |
| 28  6b8ea163-929e-447b-97a9-b0d05241ae26 | **Pain** | **Douleur** | Can the respondent explain what “pain” means to them?  *  *  *  *  *  Can the respondent give any examples of something that might cause them pain?  *  *  *  *  * |  |
| 29  8560b29a-5f92-43eb-bf08-1a91481cb6a1 | I don’t have any pain today | Je ne ressens aucune douleur aujourd’hui | Looking at the scale on the thermometer, if ‘I don’t have any pain today’ is 0, what number would you give the other response options?  *  *  *  *  * |  |
| 30  a0aedff6-401c-42f3-892c-820835b44857 | I have a little bit of pain today | Je ressens un tout petit peu de douleur aujourd’hui |  |
| 31  91cad745-e8a5-40e6-9483-9ec17fba945f | I have a bit of pain today | Je ressens un peu de douleur aujourd’hui |  |
| 32  92919ede-908d-4466-8b27-8e080c158289 | I have quite a lot of pain today | Je ressens pas mal de douleur aujourd’hui |  |
| 33  71c55240-4489-4418-ae64-bf470f6cdfe1 | I have a lot of pain today | Je ressens beaucoup de douleur aujourd’hui |  |
| 34  e7499a9b-d4f8-459c-9769-a9e0177ebffd | **Tired** | **Fatigué** | Can the respondent explain what the question means by “tired”?  *  *  *  *  * |  |
| 35  10e08334-0465-4d48-9f62-2b7174e8721d | I don’t feel tired today | Je ne me sens pas fatigué(e) aujourd'hui | Looking at the scale on the thermometer, if ‘I don’t feel tired today’ is 0, what number would you give the other response options?  *  *  *  *  * |  |
| 36  69fb97b3-480a-4163-93dc-9ddeab34fe39 | I feel a little bit tired today | Je me sens un tout petit peu fatigué(e) aujourd'hui |  |
| 37  d186cc54-fa67-4d91-a3f3-fa968ee21ccc | I feel a bit tired today | Je me sens un peu fatigué(e) aujourd'hui |  |
| 38  78b7b308-9f25-480d-be43-29339c579d92 | I feel quite tired today | Je me sens assez fatigué(e) aujourd'hui |  |
| 39  aa0264e5-81bf-4e58-995f-94c777cc94b4 | I feel very tired today | Je me sens très fatigué(e) aujourd'hui |  |
| 40  a97f42cc-c8a2-444d-a51e-92f596aa409d | **Annoyed** | **Contrarié** | Can you explain what ‘annoyed’ means?  Can the respondent give examples of times when they might be “annoyed”?  *  *  *  *  * |  |
| 41  1f7500e9-b619-42e7-8ff0-aca5c99a3d9e | I don’t feel annoyed today | Je ne me sens pas contrarié(e) aujourd'hui | Looking at the scale on the thermometer, if ‘I don’t feel annoyed today’ is 0, what number would you give the other response options?  *  *  *  *  * |  |
| 42  0073acb2-0f74-430b-a67f-07624e79e35f | I feel a little bit annoyed today | Je me sens un tout petit peu contrarié(e) aujourd'hui |  |
| 43  b8591182-f927-4f65-aa73-03f1d30a4ad4 | I feel a bit annoyed today | Je me sens un peu contrarié(e) aujourd'hui |  |
| 44  6e7b1858-b718-4bf3-86bb-6444a783f433 | I feel quite annoyed today | Je me sens assez contrarié(e) aujourd'hui |  |
| 45  727142cc-5ae0-41b1-b5ca-5fb0f66d5b30 | I feel very annoyed today | Je me sens très contrarié(e) aujourd'hui |  |
| 46  bc5606ff-b1ea-4342-b0dc-a59a0f995514 | **School Work/Homework (such as reading, writing, doing lessons)** | **Travaux/devoirs scolaires (comme la lecture, l’écriture, faire ses leçons)** | Can you give an example of a piece of work you might do at school?  *  *  *  *  *  Can you give an example of a piece of homework?  *  *  *  *  * |  |
| 47  d0f99b53-fb11-40bc-98ae-33f60a1b091d | I have no problems with my schoolwork/homework today | Je n’ai aucune difficulté à faire mes travaux/devoirs scolaires aujourd’hui | Can the respondent explain what the question means by “problems with”?  *  *  *  *  *  Looking at the scale on the thermometer, if ‘I have no problems with my schoolwork/homework today’ is 0, what number would you give the other response options?  *  *  *  *  * |  |
| 48  9a24f5d7-6ddc-405f-b73f-c69bea58c4ab | I have a few problems with my schoolwork/homework today | J’ai un tout petit peu de difficulté à faire mes travaux/devoirs scolaires aujourd’hui |  |
| 49  ea044e22-a6b7-4485-9483-89a41208dfd3 | I have some problems with my schoolwork/homework today | J’ai un peu de difficulté à faire mes travaux/devoirs scolaires aujourd’hui |  |
| 50  e43dc551-3fcf-4293-8c74-94732e18608a | I have many problems with my schoolwork/homework today | J’ai beaucoup de difficulté à faire mes travaux/devoirs scolaires aujourd’hui |  |
| 51  8db74a4e-a2ef-41a3-88f2-1ce16732a82e | I can’t do my schoolwork/homework today | Je n’arrive pas à faire mes travaux/devoirs scolaires aujourd’hui |  |
| 52  3427cd1a-2f7d-4946-93d7-8fbb421c2c07 | **Sleep** | **Dormir** | What does ‘sleep’ mean to you?  *  *  *  *  *  Can the respondent explain what the question might mean by “problems” in terms of sleeping?  *  *  *  *  * |  |
| 53  fab383ca-2bbe-48a7-a68e-e41ec5483c61 | Last night I had no problems sleeping | La nuit dernière, je n’ai eu aucun mal à dormir | Looking at the scale on the thermometer, if ‘Last night I had no problems sleeping’ is 0, what number would you give the other response options?  *  *  *  *  * |  |
| 54  754d2918-f742-44d1-bb61-25e52d46fbdf | Last night I had a few problems sleeping | La nuit dernière, j’ai eu un tout petit peu de mal à dormir |  |
| 55  60cde490-dac3-49b6-af60-fd6e8b15874e | Last night I had some problems sleeping | La nuit dernière, j’ai eu un peu de mal à dormir |  |
| 56  f3a9a727-7ef8-4be6-9b04-4f97f855dbf8 | Last night I had many problems sleeping | La nuit dernière, j’ai eu beaucoup de mal à dormir |  |
| 57  09c251df-4fb5-44b0-8c94-4e800f2bd519 | Last night I couldn’t sleep at all | La nuit dernière, je n’ai pas du tout dormi |  |
| 58  4709c690-24c7-4dd0-b9ad-e83d38606244 | **Daily routine (things like eating, having a bath/shower, getting dressed)** | **Routine quotidienne (comme manger, prendre un bain/une douche, s’habiller)** | What kind of activities would the respondent say is part of a “daily routine”?  *  *  *  *  * |  |
| 59  4eb1a3f9-3600-4c28-b66c-7e5d0f80bc95 | I have no problems with my daily routine today | Je n’ai aucune difficulté avec ma routine quotidienne aujourd’hui | Looking at the scale on the thermometer, if ‘I have no problems with my daily routine today’ is 0, what number would you give the other response options?  *  *  *  *  * |  |
| 60  0a26d0cd-1481-4fc7-8a95-68a444c886cb | I have a few problems with my daily routine today | J’ai un tout petit peu de difficulté avec ma routine quotidienne aujourd’hui |  |
| 61  63958912-e76d-4e8e-aefb-8b182313d76a | I have some problems with my daily routine today | J’ai un peu de difficulté avec ma routine quotidienne aujourd’hui |  |
| 62  7b2a5394-3cf2-44e8-bbcd-36f4b293c058 | I have many problems with my daily routine today | J’ai beaucoup de difficulté avec ma routine quotidienne aujourd’hui |  |
| 63  c80f54a0-1687-486b-b1ee-1d710449609f | I can’t do my daily routine today | Je n’arrive pas à faire ma routine quotidienne aujourd’hui |  |
| 64  e0cbb4a0-a6ce-4dbd-b50d-a656334c545f | **Able to join in activities (things like playing out with your friends, doing sports, joining in things)** | **Capable de participer à des activités (comme jouer dehors avec tes amis, faire du sport, te joindre à des activités)** | Can the respondent give examples of things they might do with friends?  *  *  *  *  *  Can the respondent give an example of a type of sport?  *  *  *  *  * |  |
| 65  0217e10b-9a8f-4cb8-987a-dc9ec029a8a5 | I can join in with any activities today | Je peux participer à toutes les activités aujourd’hui | Looking at the scale on the thermometer, if ‘I can join in with any activities today’ is 100, what number would you give the other response options?  *  *  *  *  * |  |
| 66  7834ca51-4d99-43d9-b06c-28c4c6ef2d6b | I can join in with most activities today | Je peux participer à presque toutes les activités aujourd’hui |  |
| 67  c93a118a-d579-4929-8d0e-40d71435bc70 | I can join in with some activities today | Je peux participer à quelques activités aujourd’hui |  |
| 68  9bdb7adc-8337-4ff8-a818-79c01cf1d402 | I can join in with a few activities today | Je peux participer à très peu d’activités aujourd’hui |  |
| 69  44f3b770-2618-4f3a-a649-b9186c744dab | I can join in with no activities today | Je ne peux participer à aucune activité aujourd’hui |  |

| 1. **Overview questions for the respondents** | **(2) respondents’ answers / further probing questions** | **(3) COMMENTS & DISCUSSION** |
| --- | --- | --- |
| Did you have any problems completing the questionnaire? Were the questions too detailed or complex to the point you didn’t understand what we were asking for? Which questions? | *****  *****  *****  *****  ***** |  |
| Was the French that was used throughout the questionnaire universal/understandable? | *****  *****  *****  *****  ***** |  |
| Do you have any suggestions, anything at all, to help us improve the questionnaire? | *****  *****  *****  *****  ***** |  |
| Were there any questions that were very difficult for you to understand or confusing? Which ones? What made them difficult? Any suggestions to improve them? | *****  *****  *****  *****  ***** |  |
| Were any of the instructions difficult to understand or confusing?  Did you ever find yourself skipping over instructions or rushing through them?  Do you have any suggestions on how to improve the instructions? | *****  *****  *****  *****  ***** |  |
| Did the response options make sense? Were they appropriate for the questions? | *****  *****  *****  *****  ***** |  |
